# Assembly-free quantification of vagrant DNA inserts

**DOI:** 10.1111/1755-0998.13764

**Published:** 2023-02-15

**Authors:** Hannes Becher, Richard A. Nichols

**Affiliations:** 1 Institute of Genetics and Cancer, University of Edinburgh, Edinburgh, UK; 2 School of Biological and Behavioural Sciences, Queen Mary University of London, London, UK

**Keywords:** endosymbionts, genome skimming, nuclear pseudogenes, NUMTs, NUPTs, quantification

## Abstract

Inserts of DNA from extranuclear sources, such as organelles and microbes, are common in eukaryote nuclear genomes. However, sequence similarity between the nuclear and extranuclear DNA, and a history of multiple insertions, make the assembly of these regions challenging. Consequently, the number, sequence and location of these vagrant DNAs cannot be reliably inferred from the genome assemblies of most organisms. We introduce two statistical methods to estimate the abundance of nuclear inserts even in the absence of a nuclear genome assembly. The first (intercept method) only requires low-coverage (<1×) sequencing data, as commonly generated for population studies of organellar and ribosomal DNAs. The second method additionally requires that a subset of the individuals carry extranuclear DNA with diverged genotypes. We validated our intercept method using simulations and by re-estimating the frequency of human NUMTs (nuclear mitochondrial inserts). We then applied it to the grasshopper *Podisma pedestris*, exceptional for both its large genome size and reports of numerous NUMT inserts, estimating that NUMTs make up 0.056% of the nuclear genome, equivalent to >500 times the mitochondrial genome size. We also re-analysed a museomics data set of the parrot *Psephotellus varius*, obtaining an estimate of only 0.0043%, in line with reports from other species of bird. Our study demonstrates the utility of low-coverage high-throughput sequencing data for the quantification of nuclear vagrant DNAs. Beyond quantifying organellar inserts, these methods could also be used on endosymbiont-derived sequences. We provide an R implementation of our methods called “vagrantDNA” and code to simulate test data sets.

## 1 Introduction

Nuclear genomes of most eukaryotes contain insertions of vagrant (extranuclear) DNA. Particularly common are inserts derived from organellar DNA, which are termed nuclear DNAs of mitochondrial origin (NUMTs) or nuclear DNAs of plastid origin (NUPTs) (Hazkani-Covo et al., 2010). Vagrant inserts were studied in a phylogenetic context as early as 1994 by Lopez et al. (1994), who discovered a nuclear insert of mitochondrial origin, which they called “Numt,” in several species of *Felis*. The advent of PCR and long-range PCR facilitated the study of organellar inserts, and by the mid-1990s, Zhang and Hewitt (1996b) reviewed the reports of NUMTs and identified the promises and problems of NUMTs for evolutionary analysis—NUMTs could be used to study the pace of evolution in the cell’s genomes and the progress of endosymbiosis, but also they had the potential to mislead barcoding studies (Naciri & Manen, 2010; Schultz & Hebert, 2022). While some NUMTs may be functional under selection (Vendrami et al., 2022), the majority are probably pseudogenes. Besides organelle-derived DNAs, there are also reports of extranuclear inserts derived from endosymbionts such as *Wolbachia* (Dunning Hotopp et al., 2007) and *Buchnera* (Nikoh et al., 2010).

The quantification of vagrant DNA inserts is relatively straightforward in the presence of a high-quality genome assembly. For instance, NUMTs have been identified by mapping mitochondrial genome assemblies to nuclear ones, often using blast (Bensasson, Zhang, et al., 2001; Hazkani-Covo et al., 2010; Richly & Leister, 2004). It is also possible to screen for regions with k-mer profiles resembling mitochondrial genomes (Li et al., 2019). However, despite rapid advances in sequencing and assembly technology, and the emergence of numerous large-scale genome sequencing projects such as the Earth Biogenome Project (https://www.earthbiogenome.org; Lewin et al., 2018), the Darwin Tree of Life Project (https://www.darwintreeoflife.org; The Darwin Tree of Life Project Consortium, 2022), the Vertebrate Genome Project (https://genome10k.soe.ucsc.edu; Rhie et al., 2021) and the 10,000 Plant Genomes Project (https://db.cngb.org/10kp/; Cheng et al., 2018), high-quality assemblies are available for the minority of species. In the absence of high-quality genome assemblies, the quantification of extranuclear inserts is more challenging. Fragmented genome assemblies commonly lack repetitive sequences and even assemblies which appear to be complete, or nearly so, can contain regions where repetitive sequences have been collapsed, causing the assembled length to be shorter than the actual genome size, which would bias estimates of the frequency of inserts. Thus, an assembly-free approach to quantify extranuclear inserts is desirable in the case of fragmented assemblies and to cross-verify the results from more complete assemblies.

Instead of using a nuclear genome assembly, we propose to estimate the frequency of vagrant inserts directly from sequencing reads. However, the estimation is not a straightforward case of counting the relative numbers of vagrant and extranuclear sequences. For example, in the case of NUMTs, a high-throughput sequencing data set could be mapped against a mitochondrial genome assembly. The main obstacle would then arise when it came to classifying these reads into NUMT and organellar mitochondrial categories. They might be classified according to their sequence divergence from the reference, whereby low-divergence matches could be assumed to be true mitochondrial DNA, and higher-divergence matches assumed to be derived from NUMTs. Such an approach has two obvious drawbacks: the estimate will depend on some customized divergence threshold, and, in addition, some reads that are identical to the true mitochondrial genome might actually be derived from a NUMT (they may be recent, not-yet-diverged inserts). An alternative approach would be to screen sequencing data for reads spanning NUMT insertion sites. This approach would be most effective with high-quality long sequencing reads as produced by PacBio’s circular consensus technology. This is because longer reads are more likely to contain junctions between NUMT and ordinary nuclear DNA, which makes it possible to detect NUMT sequences even if they have not yet diverged from the true mitochondrial sequence. Unfortunately, high-quality long-read sequences are still comparatively expensive to generate, and possibly prohibitively so for species with large genomes including (but not restricted to) many grasses and other monocots, grasshoppers, and newts.

As an alternative to these approaches, we propose to exploit a sampling design which uses low-coverage (<1×) high-quality short reads (also known as low-pass or genome skimming data), from multiple individuals. This type of data is commonly generated for population studies of mitochondrial and plastid DNA or for the analysis of genomic repeats. We will show that such data sets can be used to estimate the proportion of the nuclear genome that are nuclear inserts from a particular vagrant origin. The approach exploits the information that arises when the samples contain different relative proportions of the extranuclear DNA. For example, the proportion of mitochondrial reads tends to vary among samples in routine DNA extractions, which contrasts with vagrant inserts that appear at a constant stoichiometry with other nuclear DNA sequences. For this approach to work, there must be some sites that are diverged between vagrant inserts and extranuclear sequences. Despite this, large-scale similarity as observed between NUMTs and mitochondrial sequences does not pose a problem, because the approach is based on regression and not on the identification of every single insert sequence.

Grasshoppers make a good test-bed for this approach, since they are notorious for having genomes with multiple NUMTs, which complicate phylogenetic analyses (Hawlitschek et al., 2017; Song et al., 2008). One representative, the grasshopper *Podisma pedestris*, has been studied for half a century with regard to its hybrid zone (Hewitt & John, 1972; John & Hewitt, 1970). Strong selection against hybrids has been shown in laboratory experiments (Barton, 1980; Barton & Hewitt, 1981) and in the field (Nichols & Hewitt, 1988), suggesting some level of divergence between the populations. However, we still have no data on mitochondrial differentiation between the two hybridizing populations because the presence of NUMTs has made population studies almost impossible (Bensasson et al., 2000; Vaughan et al., 1999). *Podisma pedestris* is also the insect species with the largest genome size recorded (http://www.genomesize.com, accessed November 24, 2022).

As a second example, we chose to analyse data from an organism that might have a relatively smaller number of NUMTs—the parrot *Psephotellus varius*. There are reports of low NUMT content in the chicken genome, which have been extrapolated to other bird species (Pereira & Baker, 2004), although Nacer and Raposo do Amaral (2017) discovered higher NUMT contents in two species of falcon using blast searches against published genome assemblies (Zhan et al., 2013). Even in small numbers, bird NUMTs are known to be a potential source of misleading data in the genetic analysis of their mitochondrial DNA (Sorenson & Quinn, 1998).

We initially confirm our general method’s accuracy testing it on simulated and on human data, where the number of NUMTs is relatively well characterized because of the exceptionally high quality of the human genome assembly. We then proceed to quantify the NUMT content in a dedicated data set of the grasshopper *Po. pedestris* and in a re-analysis of a museomics data set of the parrot *Ps. varius* (McElroy et al., 2018).

## 2 Materials and Methods

### 2.1 Methods to estimate the proportion of extranuclear inserts

#### 2.1.1 General method

**Figure 1:**
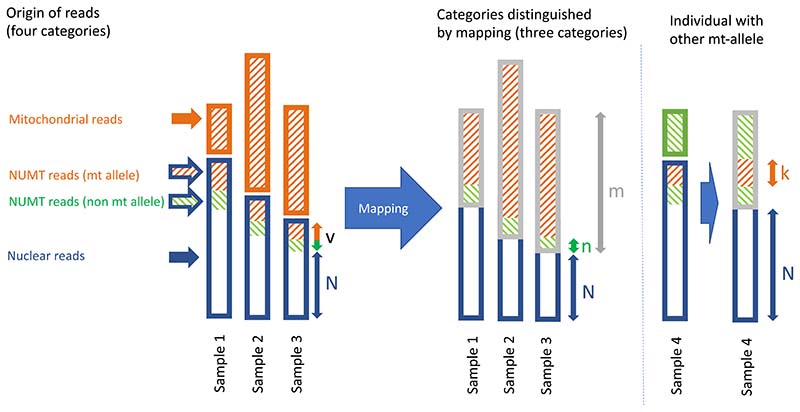
A schematic showing the proportions of reads in four different categories. In samples 1, 2 and 3, the first two categories of reads cannot be distinguished by mapping because (organellar) mitochondrial reads and NUMT reads carry the same SNP allele (as an example, the A allele). Only the *n* reads carrying an alternative allele (T, G or C) can be classified as of NUMT origin. Sample 4 had another (T, green) allele in its organellar mitochondrial genome; hence, in this case, the *k* orange reads carrying the alternative alleles (A, G or C) can be classified as NUMTs. We wish to estimate the ratio of NUMTs compared to other nuclear DNA, *v/N*, but this ratio cannot be observed directly in any one of these samples. Fraction *m* denotes all reads with sequence similarity to the mitochondrial genome.

Figure 1 shows an example in which the proportion of reads from the mitochondrial DNA (orange boxes) differs between three samples. We wish to estimate *v*/*N*, which is closely related to *v*/(*N* + *v*), the proportion of the nuclear DNA which consists of NUMTs (orange and green areas within blue boxes). We cannot observe this value directly because some NUMT reads are indistinguishable from organellar reads.

In Figure 1, the difference in the allele frequencies among the reads from different samples is due to the different amounts of organellar DNA. The observed frequency of the NUMT-specific alleles in each sample, *P*_o_ = *n*/*m*, should show a predictable relationship with the proportion of reads mapping to the mitochondrial genome, *m*/*N*. Using the notation from Figure 1, this observed frequency would be.

(1) $$P_{o}=n/m=n/N\times N/m$$

taking logs,

$$\text{log}\left(P_{o}\right)=\text{log}\left(n/N\right)+\text{log}\left(N/m\right).$$

Hence, if the underlying assumptions hold, the plot of log(*P*_o_) vs. log(*N*/*m*) should be a 1:1 line with an intercept of log(*n*/*N*). Possible deviations from these assumptions are that the frequency of the NUMT-specific alleles in the nuclear genome differs between samples, the total number of NUMT sequences in the nuclear genome differs between samples, the relative mapping rate of the different categories of read differs between samples, and that the identity of the organellar allele has been mistaken (either an outright error, or because of heteroplasmy).

For single nucleotide polymorphism (SNP) loci showing the expected relationship, the intercept, *n*/*N*, can be used as a lower bound on the value we wish to estimate (*v*/*N*) because *n* ≤ *v*. Different SNP loci will give different estimates because their NUMT allele frequency (*n*/*v*) will vary, depending on the evolutionary history of the integration of mitochondrial sequences into the nuclear genome. An SNP locus would give a precise estimate, only if *n* = *v*, which would mean that all NUMT loci had a different allele from the organellar genome.

We used a linear mixed effects model (using the lmer function from the R package lme4; Bates et al., 2015) to identify the locus with the highest intercept from a selection of loci with high values of *n*/*v*. Multiple loci were included in the regression because they provide within-sample replication, which can be used to characterize any consistent deviation of each sample from the regression. Sample-specific deviations could be due to different mapping efficiencies of that mitochondrial genome to the reference, or undetected contamination by DNA of another species. Any such effects were allowed for by fitting a random intercept for each sample. We call the estimate obtained from this regression the “intercept estimate” of *v*/*N*. To turn the logarithm of *v*/*N* obtained from the regression fit into an estimate for *v*/(*N* + *v*), we take the “inverse logit transform,” exp(*x*)/(1 + exp(*x*)).

#### 2.1.2 Mapping depth estimate

An upper-bound on *v*/*N* can be obtained simply from the lowest alignment rate (*m*/*N*) observed in any sample. In the case of Figure 1 the lowest estimate comes from Sample 1: *m*_1_/*N*_1_. This is greater than or equal to *v*/*N* because *m* ≥ *v*. It is a precise estimate only when *m* = *v*, which would mean that there were no reads from the organellar mitochondrial genome. We call this estimate (obtained again by taking the inverse logit transform) the “mapping depth estimate.”

#### 2.1.3 Diverged sites estimate

A second statistical estimate can be obtained if some individuals have different organellar mitochondrial haplotypes. This is illustrated in Figure 1 by Sample 4, whose mitochondria carry the T allele. In Samples 1, 2 and 3, the *n* reads carrying allele T, G and C are unambiguously of NUMT origin, with counts *n*_T_, *n*_G_ and *n*_C_ respectively. Similarly in Sample 4, the *k* reads of NUMT origin can be broken down into *k*_A_, *k*_G_ *and k*_C_. We cannot directly count the NUMT reads with A alleles in Samples 1, 2 and 3, but we can estimate their proportion from Sample 4 as *p*_T_ = *k*_A4_/*N*_4_. Similarly we cannot count the NUMT reads with the T allele in Sample 4, but we can estimate their proportion as *p*_T_ = Σ_*i*_(*n*_T*i*_)/Σ_*i*_(*N_i_*), where summation is over samples.

These estimates of the obscured proportions can be used to obtain an estimate of the desired quantity *v*/*N*. Consider two sets of samples A and B:

A the mitochondrial allele is *x* (*x* ∈ {T, A, G, C}), sample *i* has *v_i_* un-ambiguous NUMT reads, *m_i_* which map to the mitochondrial genome, and *N_i_* reads which do not. An estimate of the frequency of the *x* allele is obtained from the B sample as *p*_x_ = Σ_*j*_(*n_xj_*)/Σ_*j*_(*N_i_*),
B the mitochondrial allele is *y* (*y* ∈ {T, A, G, C}\{*x*}), sample *j* has *k_j_* unambiguous NUMT reads, *m_j_* reads which map to the mitochondrial genome, and *N_j_* reads which do not. An estimate of the frequency of the *y* allele is obtained from the A sample as *p_y_* = Σ_*i*_(*n_xi_*)/Σ_*i*_(*N_i_*).

The estimates of *v*/*N* for the A set is therefore.

$$p_{x}+\left(\sum_{i}v_{i}/\sum_{i}N_{i}\right),$$

and for the B set, the equivalent value is

$$p_{y}+\left(\sum_{j}k_{j}/\sum_{j}N_{j}\right).$$

We call these the “diverged sites estimates.”

### 2.2 Grasshopper DNA extraction and sequencing

We collected samples from multiple populations from either side of the *Podisma pedestris* hybrid zone. For long-term storage, hindlegs were kept in pure ethanol at 4°C. After equilibrating these in deionized water on ice for 10 min with their exoskeleton cut open, we extracted whole genomic DNA using a Blood & Tissue Kit (Qiagen). TruSeq sequencing libraries were generated, and sequencing was carried out at the Bart’s and the London Genome Centre on the NextSeq 500 platform generating paired-end reads of 76 nucleotides (nt) length.

### 2.3 Processing of sequencing data and variant calling

We downloaded forward sequencing reads of 26 human samples (Text S1), which we had selected randomly from the list of samples from the 1000 Genome Project for which there was whole-genome sequencing data available. We also retrieved parrot whole-genome sequencing data from a previous study (McElroy et al., 2018); the data sets are listed in Text S1. We downloaded from GenBank a human mitochondrial reference (NC_012920.1) and we assembled, de novo, the mitochondrial genome sequences for the grasshopper and parrot using novoplasty (Dierckxsens et al., 2016) and getorganelle (Jin et al., 2020), respectively. We annotated the grasshopper mitogenome via the MITOS web server (Bernt et al., 2013).

The subsequent steps were identical for all data sets. We mapped high-throughput sequencing reads with bwa (Li & Durbin, 2009) to the appropriate mitochondrial genome assemblies creating BAM files and retaining unmapped reads. Using samtools (Li et al., 2009), we then sorted the alignment files and generated per-sample mapping statistics from which we extracted the total amount of read data and the amount of base pairs mapped (taking into account soft-clipping). We marked duplicates with picard tools (http://broadinstitute.github.io/picard/). We then called single-nucleotide variants with freebayes (https://arxiv.org/abs/1207.3907) across all BAM files per species. To retain apparent variants caused by NUMTs, we ran freebayes with the command line parameters “--haplotype-length -1 --min-alternate-fraction 0.01 --min-alternate-count 2 --pooled-continuous -p 1 -X -u -I,” counting the number of occurrences of any nucleotide at any site in the alignments.

From the resulting VCF files (one per species), we extracted all nucleotide allele counts for each variant site by means of an interactive python script which uses the scikit-allel package (version 1.3.2, https://github.com/cggh/scikit-allel). In each individual and at each site, the most abundant allele was designated the genotype.

### 2.4 Analyses of variant data and implementation of the method

We excluded individuals with excess missing data. Our methods described above are implemented in R with the intercept method relying on a mixed-effect modelling approach, which limits the number of variant sites that can be analysed on a desktop computer. This method thus selects, per data set, 400 of the most informative SNPs, by subsampling using a weighting proportional to each SNP’s average allele frequency (*P*_o_). This selection minimizes the bias in the estimate, since these SNPs have the highest proportion of distinguishable reads (*n*/*v*; Figure 1). Our 95% confidence intervals are based on the standard deviation of the intercept estimates (“standard error” in R’s terminology); they correspond to 1.96 standard deviations on either side of the respective intercept estimate, transformed back into linear space.

To select sites for the diverged sites method, we carried out principal component analyses (PCAs) on the variant data. In both the parrot and grasshopper data sets this PCA identified two populations, arbitrarily designated populations A and B, carrying distinct mitochondrial genotypes. We then selected sites that showed fixed genotype differences between these populations to obtain the diverged sites estimates as described above.

### 2.5 Simulations

To test the intercept method, we generated a simulation pipeline that can be run locally on a desktop PC. The pipeline generates a random genome sequence whose length can be specified. It also generates a random extranuclear sequence 16,000 nt in length. Then, over the course of 15,000,000 “years,” it is checked each year whether the extranuclear DNA experiences a nucleotide substitution (at a rate typical for insects) and whether an insertion event happens (at a rate that can be specified). Then the nuclear and extranuclear genomes are written out. After that, sequencing reads are simulated from the nuclear genome using wgsim (https://github.com/lh3/wgsim), adding varying amounts of extranuclear DNA (per-sample proportions drawn from an exponential distribution with a mean of 1%). The reads are then mapped using bwa-mem2 (Vasimuddin et al., 2019) and variants are called with freebayes. The resulting VCF files are processed as described above.

Using this pipeline, we simulated three nuclear genome sizes, 250, 500 and 1000 Mb, and seven insertion rates, 0.00001–0.00004 insertions per year (in steps of 0.000005). We simulated 10 replicates for each parameter combination and recorded how many times the proportion of nuclear inserts observed in each simulation was within the confidence interval returned by the intercept method. We then fitted a binomial-family generalized linear model using the log-transformed values of “genome size in Mb” and “expected mapping depth of nuclear reads to insert sequences” as predictors. To assess the effect of variable insertion rates along the extranuclear sequence, we also ran simulations where the start location of the insert sequence (extrapolated to the length of the extranuclear DNA) was drawn from an asymmetric unimodal beta distribution (shape parameters set to 3 and 2).

## 3 Results

### 3.1 The relative amounts of extranuclear and insert DNA

**Figure 2:**
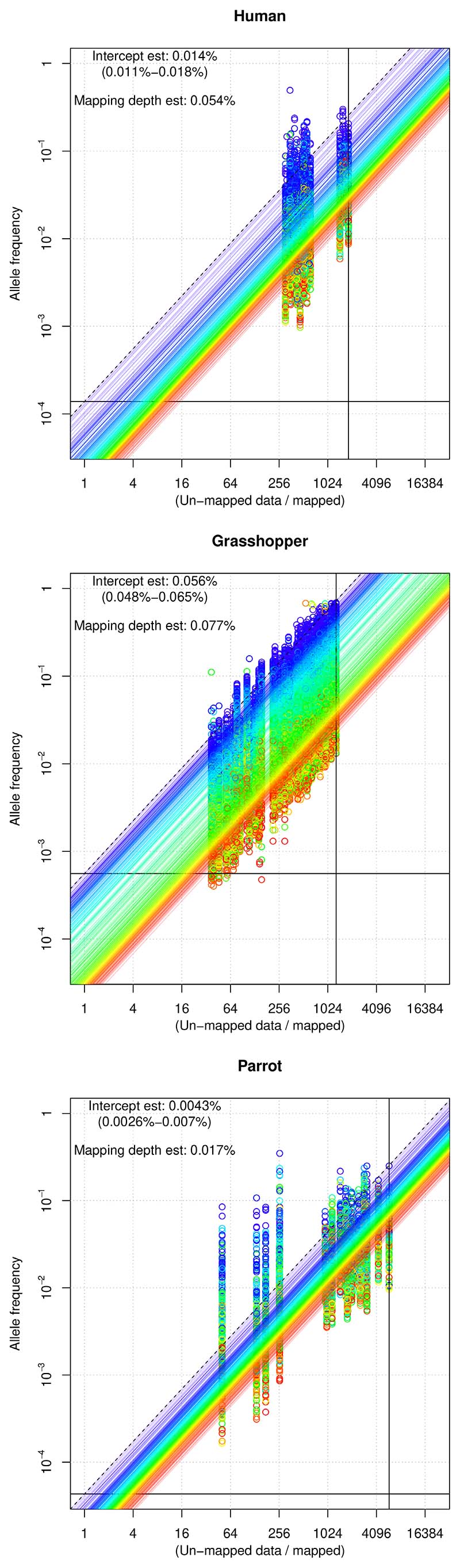
Change in the frequency of NUMT alleles with the mitochondrial mapping ratio (unmapped/mapped). The raw data are plotted as circles. The vertical stacks of points represent frequencies of different loci from the same sample, each locus being given a different colour (from red to violet according to the global average allele frequency). The lines with the corresponding colour show the best fit to Equation 1. Both axes are logarithmic. The intercept on the log scale will correspond to *x* = 1 (half the reads mapping to the mitochondrial genome, log(1) = 0). The two solid lines correspond to the minimum proportion of reads mapping to the mitochondrial genome across all samples (vertical) and to the intercept of the SNPs with the highest fitted allele frequency (horizontal). The dashed line shows the fitted relationship for the locus with the highest estimated allele frequency. The values of the horizontal solid and dashed lines at *x* = 1 are both estimates of the proportion of the genome composed of NUMTs (intercept estimate).

Across all three species and individuals analysed, the proportion of the data that could be aligned successfully to the appropriate mitochondrial reference was <3%, indicating that the largest part of each (cleaned) data set comprised nuclear DNA. Mitochondrial variant calling within each individual sample revealed highly variable allele frequencies in different individuals, consistent with different ratios of NUMTs to mitochondrial DNA. The allele frequencies at specific sites were correlated among samples and covaried with the proportion of the data that could be aligned to the mitochondrial genome (diagonal lines in Figure 2). This linear relationship is predicted by Equation 1: because most of the variation was due to differences in the proportion of mitochondrial DNA in different samples (rather than differences in allele frequencies in the NUMTs), samples with more mitochondrial DNA had lower NUMT allele frequencies and vice versa.

### 3.2 Human data

To validate our intercept method, we ran it on 26 human samples from the 1000 Genomes project. Assuming a genome size of 3.5 Gb, these data corresponded to genomic coverage depths ranging from 0.28× to 1.8× (median of 0.65×). The alignment rates of reads to a human mitochondrial reference had a bimodal distribution with four samples showing extremely low rates close to 0.05%. The greatest intercept (dashed line in Figure 2) generated an estimate for the NUMT composition of the nuclear genome of 0.014% (95% confidence interval: 0.011%–0.018%). The estimate based on the lowest proportion of mapped reads (“mapping depth estimate,” solid line) was 0.054%.

### 3.3 Grasshopper data

Our de novo assembly of the *Podisma pedestris* mitochondrial genome was 16,008 bp in length. It contained 37 genes: two rRNAs, 13 protein-coding genes and 22 tRNAs. The highly AT-rich D-loop region contained a direct repeat of 383 bp. In total 14,664 bp of the assembly was made up of genes, corresponding to 91.6% of its length. There were 11,176 sites in coding regions, 1416 of which were four-fold degenerate. Overall, the *Po. pedestris* mitochondrial genome is similar in size and structure to those of other animals (Boore, 1999).

**Figure 3:**
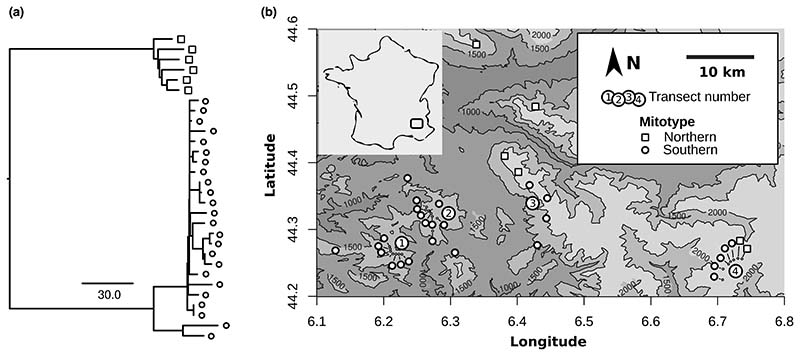
Sequence relationships and spatial distribution of grasshopper mitotypes. (a) A dendrogram based on (true) mitochondrial sequence polymorphism between samples of the grasshopper *Podisma pedestris*. The scale bar indicates the number of substitutions per mitochondrial genome. The symbols match the legend in (b). (b) The locations of the sampling sites and the distribution of two diverged mitotypes. The inset shows the approximate position of the study area in France.

The overall data sequenced for each individual corresponded to genomic mapping depths of 0.056–0.34× (median 0.069×). The mapping rates to the mitochondrial reference varied considerably between samples, ranging from 0.08% to 2.6%, reflecting varying ratios of nuclear DNA to (true) mitochondrial DNA among samples. A PCA revealed that the samples clustered in two groups (mitotypes) as we expected from the known chromosomal polymorphism in the species. After excluding individuals with low mapping depth, where genotype calls might have been confounded with NUMT variants, we identified 111 sites that showed mitochondrial variants with fixed allelic differences between two groups, corresponding to a divergence of 0.69% (Figure 3). Using Brower’s (1994) rate of 2.3% pairwise divergence per million years resulted in a crude estimate of 300,000 years for the divergence between the two populations.

**Figure 4:**
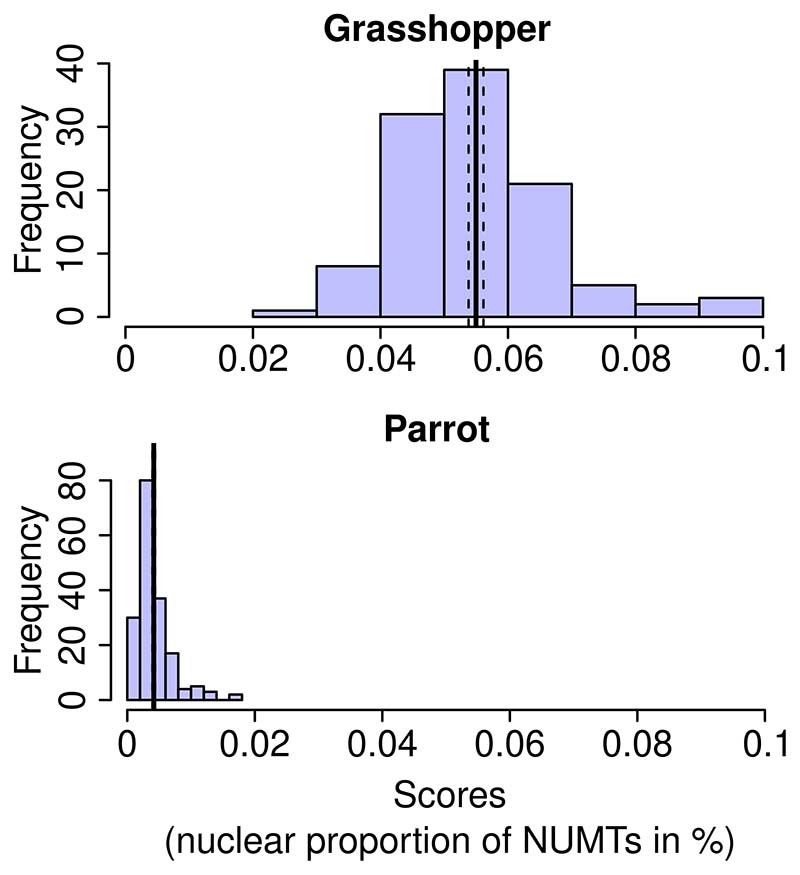
Insert quantification in diverged populations. Within-individual allele frequencies are shown for sites with fixed mitochondrial differences between two populations of the grasshopper *Podisma pedestris* (top, 111 sites) and the parrot *Psephotellus varius* (bottom, 178 sites). Both histograms share the same *x*-axis. Fat vertical lines indicate the means, and dashed lines the associated standard errors.

Our estimates for the nuclear proportion of NUMTs were 0.056% (95% confidence interval [CI]: 0.048%–0.065%) (intercept estimate) and 0.077% (mapping depth estimate). Because there were 111 SNPs with fixed differences between the northern and southern populations, we could additionally obtain a diverged sites estimate. Figure 4 shows the allele frequency scores for 111 loci resulting in an overall estimate of 0.055% (*SE* 0.0012%).

### 3.4 Parrot data

As reported by McElroy et al. (2018), the parrot data set contained considerable amounts of adapter sequences, ranging from 4% to 41% of the data, which we excluded in order to generate more accurate abundance estimates. Our de novo assembly of the parrot mitochondrial genome, generated from sample SRR6214434, was 19,278 bp in length. The per-sample mapping rates varied over two orders of magnitude, from 0.02% to 1.9%. A PCA run on individual mitochondrial alleles separated the samples into two groups corresponding to the phylogroups inferred by McElroy et al. (2018). The intercept estimate of the nuclear proportion of mitochondrial inserts was 0.0043% (95% CI: 0.0026%–0.070%) The mapping depth estimate was 0.017%. As with the grasshopper data, we analysed the minor allele frequencies at each site that showed fixed mitochondrial difference between the populations (Figure 4). This resulted in a diverged sites estimate of 0.0033% (*SE* 0.00016%).

### 3.5 Simulations

**Figure 5:**
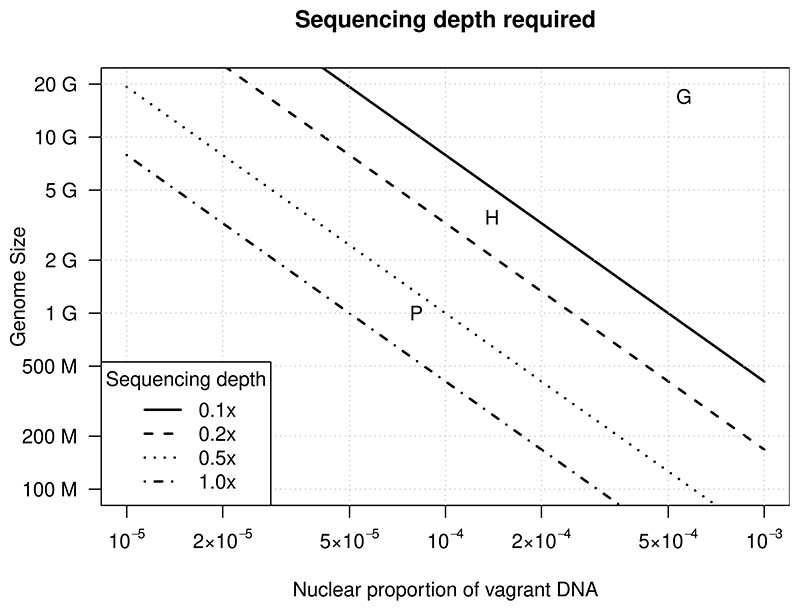
The effect of sequencing depth, nuclear proportion of vagrant DNA and nuclear genome size on the accuracy of the intercept estimate for vagrant DNA derived from a 16-kb genome. The isolines indicate 95% estimation accuracy (19 out of 20 fits successful and accurate). Different line styles indicate the different levels of sequencing depth required to achieve 95% estimation accuracy depending on genome size and vagrant DNA ratio. The letters indicate the three data sets analysed in this study (P, parrot; H, human; G, grasshopper).

Running the intercept method on simulated data allowed us the assess the expected accuracy depending on the nuclear genome size and the expected mapping depth to the extranuclear reference due to insert sequences. The model fit is summarized in Table 1 and Figure S1. We then used this model to predict whether a data set is sufficient to generate an accurate intercept estimate. Figure 5 summarizes the effects of genome size, vagrant DNA proportion and sequencing depth on estimation accuracy. At constant accuracy and sequencing depth (i.e., following one of the isolines in Figure 5) there is a trade-off between nuclear genome size and proportion of vagrant DNA that can be detected. All the data sets we analysed (letters in Figure 5) are in a region of the parameter space where high accuracy is expected.

We also ran simulations with insertion rates varying along the extranuclear reference. It turned out that the intercept method was robust to these. Further information on how to deal with potentially unequal insertion rates are given in the tutorial in the Supporting Information.

## 4 Discussion

In this study, we have introduced a general statistical method for estimating the nuclear proportion of DNA inserts of extranuclear origin. We validated this method by re-estimating the nuclear proportion of NUMTs in humans and then analysed data from the grass-hopper *Podisma pedestris* and the parrot *Psephotellus varius*. Because the grasshopper and parrot data sets each contained individuals from two diverged populations, we were able to obtain an additional estimate of NUMT abundance. After discussing our results, we will address the robustness and utility of our methods.

### 4.1 Estimates of NUMT abundance

Numerous studies have made use of public genome assemblies and have estimated in these assemblies the nuclear proportion of NUMTs. Estimates of the proportion of NUMTs in the human genome range from 0.0087% (Hazkani-Covo et al., 2010) and 0.0096% (Richly & Leister, 2004) to 0.012% (Bensasson, Zhang, et al., 2001) and 0.016% (Woischnik & Moraes, 2002). Our (upper-bound) mapping depth estimate was 0.056%. The intercept estimate of 0.014% falls inside the range of previous estimates, confirming the accuracy of our approach. Furthermore, our analyses of simulated data, where the nuclear proportion of NUMTs was known, allowed us to assess in which region of the parameter space accurate estimates are possible (Figure 5). All our data sets fell into regions where high accuracy is expected.

Our estimate for the nuclear proportion of NUMTs in the grasshopper *Po. pedestris* was 0.056%, which may at first sight seem low given that a number of previous reports, based on PCR and cloning studies, inferred a large number of nuclear inserts in this species (Bensasson et al., 2000; Bensasson, Petrov, et al., 2001). However, considering the species’ enormous genome size of 16.93 pg (Westerman et al., 1987), which corresponds to 16.6 Gb (Doležel et al., 2003), the *Po. pedestris* NUMT content amounts to 8.4 Mb or the equivalent of over 500 whole mitochondrial genomes inserted into the nuclear genome. Our second estimate based on sites with fixed differences between the grasshopper populations was very close—0.055%.

For the parrot *Ps. varius*, we obtained an intercept estimate of 0.0043%. While the number of NUMTs in birds was thought to be generally low with a nuclear proportion in chicken estimated to be 0.00078% (Pereira & Baker, 2004), more recent reports give a more differentiated picture with high numbers observed in certain songbirds (Liang et al., 2018) and falcons (Nacer & Raposo do Amaral, 2017). The diverged sites estimate was 0.0033%.

### 4.2 Robustness of the approach

Our general approach produced an estimate comparable to previous estimates of the human genome’s NUMT proportion. Because our methods rely on replicated data, they are less susceptible to the idiosyncrasies of individual samples, such as contamination with nonspecific DNA sequences. Making use of biological replication is also beneficial because inserts of extranuclear DNA have been shown to be polymorphic within populations and species (Ricchetti et al., 2004; Woischnik & Moraes, 2002). Harnessing biological replication is thus likely to give less biased estimates. Because our methods are designed with low-coverage sequencing data in mind, the use of multiple samples reduces the effect of stochasticity in allele sampling (assuming that the sampling error is independent among samples).

The mapping depth estimate, which is based on the individual with the lowest alignment rate, is inflated by the smallest proportion of extranuclear DNA found in the set of samples. Consequently, it should be considered a crude upper bound most useful as a cross-validation check, unless the samples were deliberately depleted for extranuclear DNA.

The diverged sites estimate is, in theory, more accurate than the intercept estimate, because it does not require the assumption that the NUMTs have a high frequency of some SNP alleles that are different from the mitochondrial reference. It does make different assumptions, in particular that the number and genotypes of the NUMTs in the A and B populations are similar. In applying these methods to other species and other vagrant genomes, it would seem prudent to obtain both the intercept and diverged sites estimate where possible. The diverged sites estimate should be the same or slightly higher (within the precision of the estimates’ confidence intervals). This cross-validation serves to check for substantial violations of the underlying assumptions. This check will be especially effective in studies with substantial divergence among extranuclear genotypes such as that between the A and B mitochondrial haplotypes in our samples, so the diverged sites estimate has low standard error.

If the sequencing platform used for data generation has a GC bias (Ross et al., 2013), this could affect the ratio of insert DNA and other nuclear DNA sequences and may thus bias our method’s estimate. We noticed higher allele frequencies in six grasshopper samples that we had sequenced with an older version of the Illumina NextSeq chemistry. However, exclusion of these samples did not change our estimates qualitatively (data not shown). With the current trend to PCR-free sequencing libraries and improving sequencing technologies such biases may be less of a concern in the future.

Contamination with nonspecific sequences can affect our estimates because our method implies nonaligned sequence to be part of the nuclear genome. This is not of concern in our data sets, because in the data sets we analysed, mito-like sequences accounted for <3% of the data. As for any bioinformatics project, it is advisable to subject the original data to standard quality assurance measures such as fastqc (https://www.bioinformatics.babraham.ac.uk/projects/fastqc/) and removal of sequencing adapters.

When estimating the abundance of nuclear inserts derived from more complex genomes, it may be useful to restrict the analysis to a subset of the variant data depending on genome features. For instance, plastid genomes tend to contain a large inverted repeat region (Kolodner & Tewari, 1979), where sequence alignment and variant calling may be unreliable. In addition to regions excluded due to prior knowledge, regions can be excluded if they show aberrant signals. Because our method is based on regression, an analysis of the residuals may be used to uncover individuals or genome regions showing unexpected patterns. To this end, we have implemented in our R package an intercept–position–plot function (see tutorial in the Supporting Information).

Our method relies on multiple samples with varying ratios of true mitochondrial DNA to nuclear DNA. We found that sufficient variation (several fold) arises in the standard use of commercial extraction kits. However, this variation could be accentuated by deliberately choosing tissues with different mitochondrial density, or modifying the extraction process (Lansman et al., 1981; Macher et al., 2018; Zhang & Hewitt, 1996a).

Finally, we would like to point our that approach is robust to the existence of nuclear vagrant DNAs that are identical in sequence to their extranuclear original DNAs. This is because, different from other methods, we do not directly identify all copies of vagrant DNA, which would not be possible with low-coverage (<1×) sequencing data. Instead, our methods are based on high allele frequencies, as would be observed at a site that recently experienced a nucleotide substitution in the extranuclear genome—leading to (near) perfect divergence between nuclear inserts and extranuclear DNA at that site.

### 4.3 Utility of the approach

Our method is applicable to a wide range of species and sources of extranuclear DNA. Because it is based on allele frequencies rather than the length of sequence inserts, knowledge of part of the extranuclear genome is sufficient for our method to work. This should be particularly useful where extranuclear genomes are difficult to assemble, as in the case of plant mitochondria (Mower et al., 2012). In addition, parts of the reference containing repeats such as the inverted repeat region on land plant plastids (Wicke et al., 2011) can be excluded, which might otherwise confound variant calling.

Because our method relies on low-coverage sequencing, it is also suitable for museum samples, which often contain degraded DNA and where contaminations may complicate analyses that target specific marker loci. Low-coverage sequencing or “genome skimming” (Straub et al., 2012) data are straightforward to generate at comparatively low cost. Different from marker-based approaches such as PCR/Sanger-sequencing or target capture, no prior knowledge about the samples’ genomes is required. The analysis of low-coverage data is also less computationally demanding than the generation of a high-quality genome assembly. In addition, population-level low-coverage sequencing data naturally lend themselves to population or landscape genetic analyses because it is usually possible from such data to reconstruct rDNA mitochondrial and (in plants) plastid sequences (Dodsworth et al., 2015; Twyford & Ness, 2016).

## Supplementary Material

Supple file

## Figures and Tables

**Table 1:** Summary of the simulation results.

	Estimate	*SE*	z-value	p-value
Intercept	3.6314	2.4267	1.496	.135
log(GS)	-1.5034	0.4217	-3.565	**.000364**
log(numtDep)	6.7679	0.9332	7.252	**4.11e-13**

*Note*: The accuracy of the intercept estimate (binary outcome) was modelled in a binomial-family generalized linear model using the host nuclear genome size, “GS,” and the mapping depth of nuclear reads to the vagrant DNA reference, “numtDep,” as (log-transformed) predictors. The model coefficients (column “Estimate”) are given on logit scale. Bold values indicates significance at the 5% level.

## Data Availability

Raw sequence reads are deposited in the SRA (BioProject PRJNA806454; see Supporting Information). The scripts required to process and analyse these data are available from the GitHub repository https://github.com/SBCSnicholsLab/pseudogene_quantification, which also contains an R package called “vagrantDNA” to make available the functions needed to carry out the analysis. This repository also contains the grasshopper and parrot mitochondrial genomes along with their annotations. A frozen version of this repository is available on Zenodo at https://doi.org/10.5281/zenodo.7576184. The simulation code is available on the GitHub repository at https://github.com/SBCSnicholsLab/vagrantDnaSim. The SRA identifiers of the human and parrot data used are listed in the Supporting Information.
