# Supplementary material for: Assembly-free quantification of vagrant DNA inserts
Source: Mol Ecol Resour. Author manuscript; Available in PMC 2024 Dec 9. (PMC7617115; doi:10.1111/1755-0998.13764)
Supplement: Supple file [file EMS179234-supplement-Supple_file.docx]

**Supplemental Information for:**

**Assembly-free quantification of vagrant DNA inserts**

Hannes Becher, Richard A. Nichols

**Table of Contents:**

| **1 Tutorial: The vagrantDNA Package** | Page 1 |
| --- | --- |
| **2 Human data** | Page 10 |
| **3 Grasshopper data** | Page 11 |
| **4 Parrot data** | Page 13 |
| **5 Figure S1 – Simulation results** | Page 14 |

# 1 The vagrantDNA package – A tutorial

The R package ‘vagrantDNA’ was developed to estimate the nuclear proportion of vagrant DNAs such a NUMTs (nuclear inserts of mitochondrial origin) from population-level low-coverage sequencing data (<1x per individual). The package can be downloaded from GitHub, where the latest updates and instruction are available: <https://github.com/SBCSnicholsLab/pseudogene_quantification>

## 1.1 Setup

In R, install devtools (if required):

# Install devtools from CRAN

install.packages("devtools")

Then, install vagrantDNA from the GitHub release file. At the time of writing, this is version 1.1.0:

# Install vagrantDNA from GitHub

devtools::install_url("https://github.com/SBCSnicholsLab/pseudogene_quantification/releases/download/v1.1.0/vagrantDNA_1.1.0.tar.gz")

# Load package

library(vagrantDNA)

The package ships with two sample datasets, humanDF and parrotDF, that were generated from public data. A third (grasshopper) sample dataset is available online. It was too large to be included in the package. To retrieve the grasshopper data, run in R:

download.file("https://tinyurl.com/4mtrbkzc", destfile = "hopper.csv")

hopperDF <- read.table("hopper.csv")

To recreate the results shown in the paper, follow the examples on the help page of ?humanDF.

## 1.2 Example – estimating the nuclear abundance of NUMTs in parrots

The main function of the package is rainbowPlot(). To estimate the NUMT proportion in the parrot (*Psephotellus varius*) genome, run:

rainbowPlot(parrotDF, seed=12345)

The seed argument is not required. This is used here to make sure the user can generate the same plot as is shown here. The text output should be:

# Intercept based on 400 SNP loci

# Estimate: 8.06e-05

# Confidence Interval: 4.83e-05 - 0.000135

# Mapping depth estimate: 0.00017

# Function call

# rainbowPlot(parrotDF, seed = 12345)

The estimate and 95%-confidence interval refer to the ‘intercept estimate’ (explained in more detail in the main text). The ‘mapping depth estimate’ is an additional, upper-bound estimate. It should always be higher than the intercept estimate. If it is not, rainbowPlot() will produce a warning.


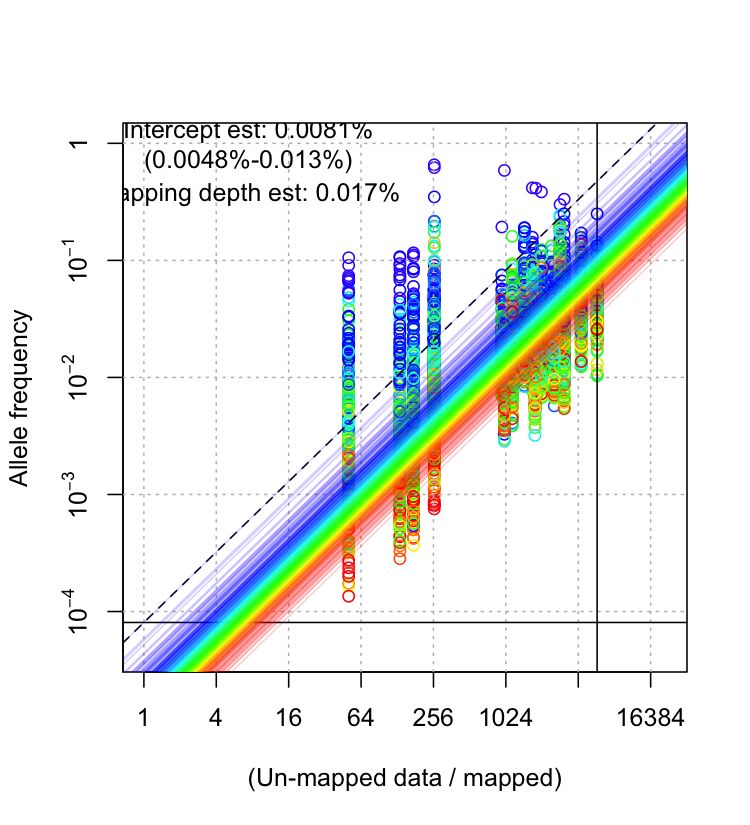


## 1.3 Assessing the model fit – Diagnostic plots

### 1.3.1 Intercept-position-plot

The intercept estimate is based on the SNP with the highest allele frequency across all samples. This assumes that divergent SNP alleles correspond to nuclear inserts of vagrant DNAs, and that the highest allele frequency is observed when all copies of a nuclear insert have a different allele to the corresponding extranuclear sequence (for instance due to a relatively recent substitution in the extranuclear sequence). This assumption may be violated, for instance if there is heteroplasmy or if there are mapping artefacts as may occur at the start or end of a reference sequence. The interceptPositionPlot() function can be used to detect such anomalies.

Running rainbowPlot() on the shipped humanDF data produces a relatively high estimate.

humanFit <- rainbowPlot(humanDF, seed=12345)

There is also a warning that the mapping depth estimate is higher than the intercept estimate. The plot looks suspicious – the SNP with the highest intercept (dashed line) is far away from all other SNPs (set of parallel lines further down). This suggests the top SNP is an outlier.


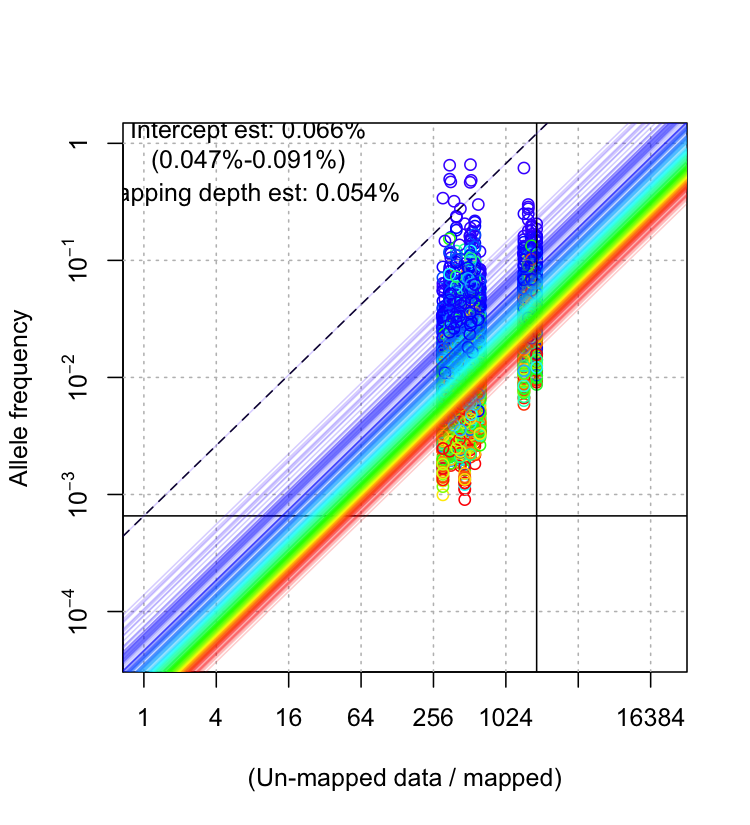


Generating a diagnostic plot, it is clear there is one outlier locus.

interceptPositionPlot(humanFit)


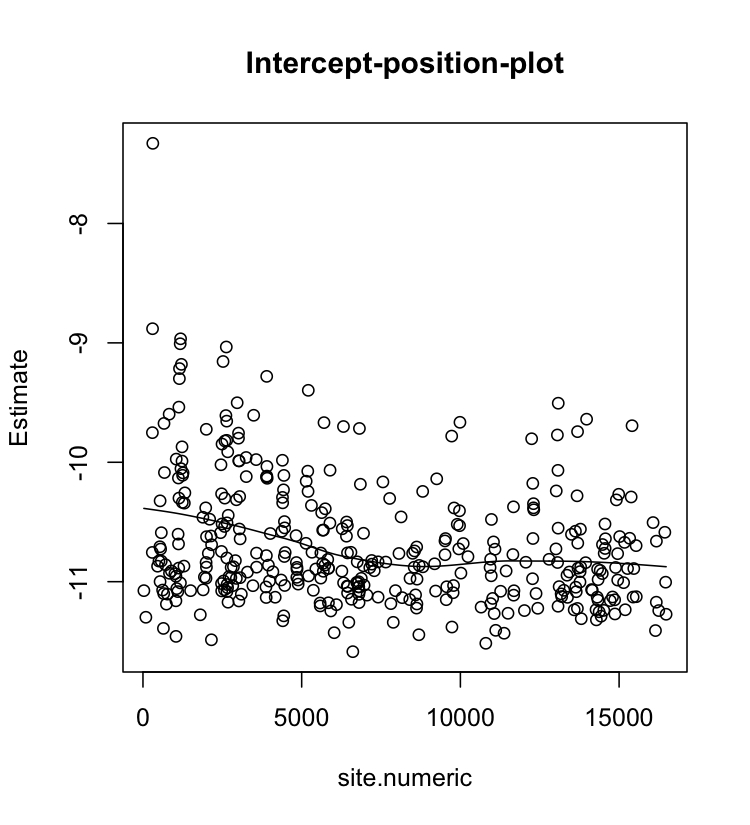


This SNP’s regression line has an intercept of around -7, much higher than all other selected SNPs. There are now two ways to deal with this. One way is to set the maxFreq argument in rainbowPlot() to a relatively low value such as 0.1. This produces a more sensible estimate in-line with previous estimates from humans:

humanFit2 <- rainbowPlot(humanDF, seed=12345, maxFreq=0.1)

Alternatively, one can also remove the offending SNP. The intercept values can be extracted from the model fit. We will look only at the first column of the model fit coefficient table:

sort(coef(humanFit$lmer.model)[[1]][1,], decreasing = T)

The output should look like this (truncated):

(Intercept) Position310 Position302 Position1185 Position1173

ERR013101 0.2305222 -7.328861 -8.881315 -8.965764 -9.007332

Position2625 Position2523 Position1211 Position1151 Position3898

ERR013101 -9.034406 -9.156001 -9.179887 -9.215314 -9.280496

The offending SNP is at position 310 and may be removed:

humanDF02 <- humanDF[humanDF$Position != 310,]

The fit and diagnostic plot look much better now – no stray lines/dots.

humanFit3 <- rainbowPlot(humanDF02, seed=12345)


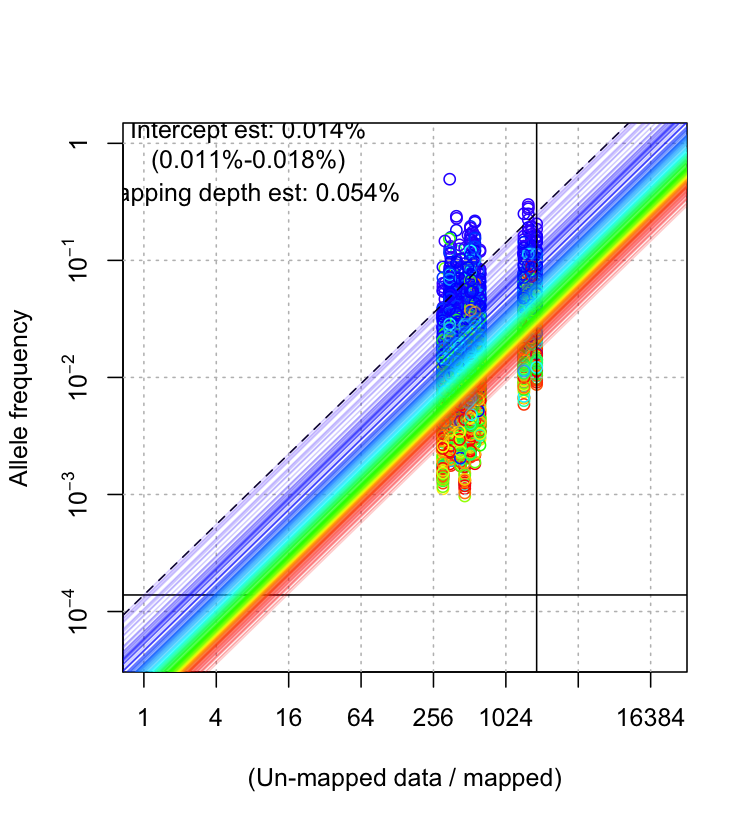


interceptPositionPlot(humanFit3)


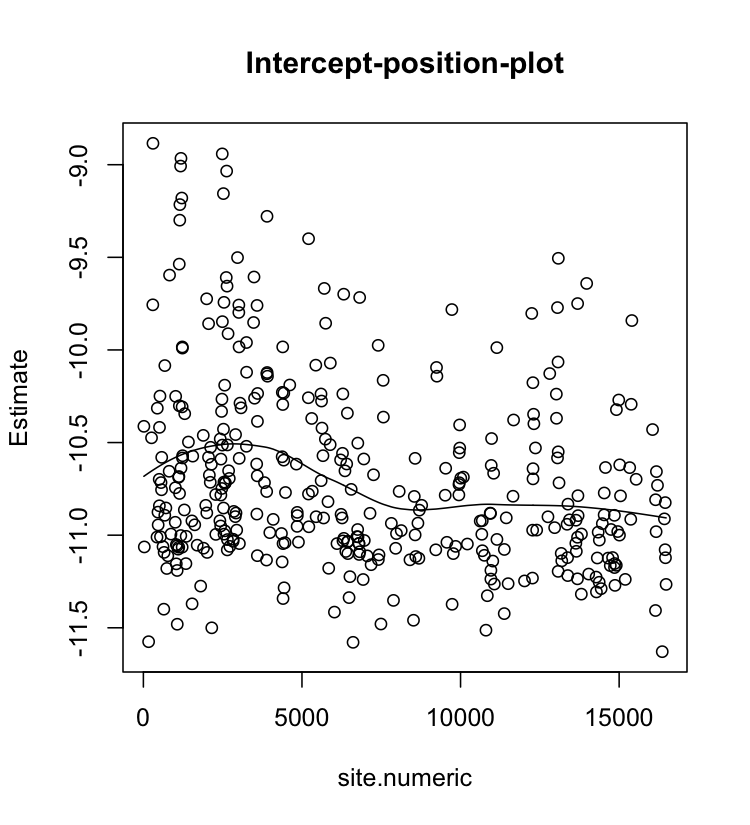


### 1.3.2 Selected-allele-plot

There is a second kind of diagnostic plot, which allows the user to test the suitability of the data set. The intercept estimate is based on alleles diverged from the extranuclear reference. Based on simulations, there must be variation in these frequencies and the majority of sample must have a median alternate allele frequency of at least 2. Otherwise the fit (if any) is likely unreliable and rainbowPlot() will give a warning.

To visualize the alternate allele counts per sample, one can use the function selAllPlot(). This can be run on the original dataset to show the distribution of alternate alleles for all sites. Alternatively, a model fit can be added as an additional input in which case the alternate allele frequencies of the chosen (high-frequency) SNPs are shown.

selAllPlot(parrotDF) # not shown here

parrotFit <- rainbowPlot(parrotDF, seed=12345)

selAllPlot(parrotDF, parrotFit)


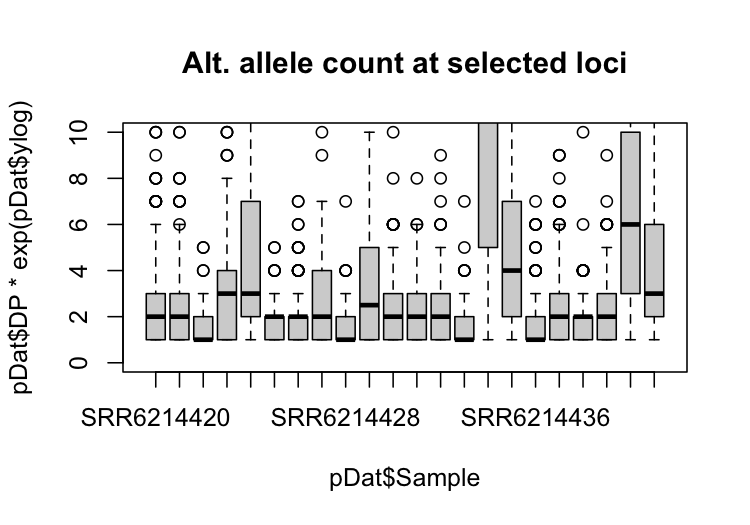


By default, the y-limits of this plot are restricted to c(0,10). This is so the user can tell whether the median allele frequencies (fat horizontal lines) are at least 2 in most samples. To inspect the full distributions, one can run:

selAllPlot(parrotDF, parrotFit, ylim=NULL)

or

selAllPlot(parrotDF, parrotFit, ylim=NULL, log='y')

Additional keyword arguments are passed to the plot() function. Is it thus possible to adjust axes labels, captions, etc.

## 1.4 If there are diverged populations – diverged-sites-estimate

If the user data set contains two populations that are diverged with respect to their extranuclear DNA, such a two mito-races, there is an additional estimate possible. This is based on the minor allele frequencies (within individuals) at sites that show fixed difference between to populations. These minor alleles are presumed to be caused by nuclear inserts.

There are two such datasets that ship with the vagrantDNA package, hopperFX and parrotFX.

To carry out a diverged-sites-estimate, run:

divEst(hopperFX)

The output will be:

# [1] Mean of A Scores: 0.00055 (SE 1.2e-05 )

# [1] Mean of B Scores: 0.000564 (SE 1.2e-05 )


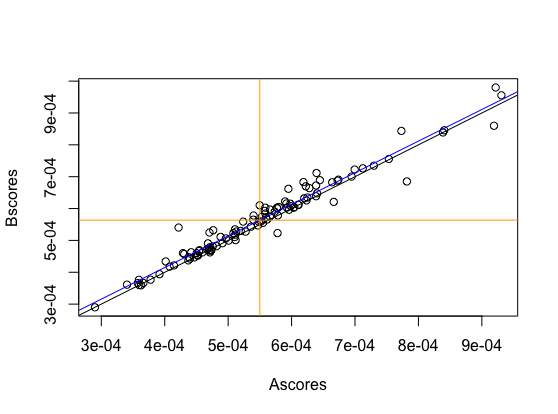


The orange lines indicate the estimates (mean A and B scores). The black line is the expected relationship between the scores (slope=1 and intercept=0). The blue line shows the regression line of best fit.

The A and B scores are retuned by divEst() and can thus be inspected or re-analysed.

divHopper <- divEst(hopperFX)

str(divHopper)

plot(divHopper$Ascores, divHopper$Bscores)

## 1.5 Data formats

The input formats required by rainbowPlot() and divEst() are described in these functions’ help pages. They are data frames (stored as CSV) containing a specific set of names columns. The GitHub repo <https://github.com/SBCSnicholsLab/pseudogene_quantification> contains code to generate such data frames from VCF files generated with Freebayes (see subfolders of the data folder).

# 2 Human data

We used forward reads of 26 datasets from the 1000 Genome project. This is a list of SRA identifiers.

| ERR251013 |
| --- |
| ERR015526 |
| SRR101474 |
| ERR013101 |
| ERR015522 |
| ERR015525 |
| ERR162841 |
| ERR013103 |
| ERR015531 |
| ERR018423 |
| SRR043397 |
| ERR019907 |
| ERR016162 |
| ERR015484 |
| ERR018436 |
| ERR016118 |
| ERR016137 |
| ERR016064 |
| ERR016081 |
| ERR018448 |
| SRR792951 |
| ERR013120 |
| ERR016251 |
| ERR018469 |
| ERR016258 |
| ERR016269 |

# 3 Grasshopper data

A list of SRA identifiers corresponding to the grasshopper data generated for and analysed in this study.

| **SRA run** | **sample** | **Lat** | **Long** | **Mitotype** | **Mappingrate** | **xCoverage** |
| --- | --- | --- | --- | --- | --- | --- |
| **SRR18000085** | 19T | 44.27062 | 6.20967 | NA | NA | NA |
| **SRR18000086** | 18T | 44.26341 | 6.22698 | F | 0.00184071 | 350.716642 |
| SRR18000087 | 17T | 44.26385 | 6.22264 | F | 0.00237417 | 564.155672 |
| SRR18000088 | 16T | 44.32815 | 6.26308 | F | 0.00175312 | 177.646677 |
| SRR18000089 | 15T | 44.32592 | 6.27052 | F | 0.00082612 | 106.313593 |
| SRR18000090 | 9T | 44.33321 | 6.26421 | F | 0.00176209 | 157.891804 |
| SRR18000091 | 8T | 44.256414 | 6.73596402 | U | 0.00163682 | 213.609945 |
| SRR18000092 | 7T | 44.256828 | 6.73064302 | U | 0.00237996 | 137.22064 |
| SRR18000093 | 6T | 44.254923 | 6.72341497 | F | 0.00189199 | 60.5464768 |
| SRR18000094 | 5T | 44.252517 | 6.71919803 | F | 0.00123144 | 52.5087456 |
| SRR18000095 | 4T | 44.252517 | 6.71919803 | F | 0.00135909 | 69.0922039 |
| SRR18000096 | 14T | 44.32701 | 6.26702 | F | 0.00087599 | 232.43903 |
| SRR18000097 | 46U | 44.2686111 | 6.12805556 | F | 0.02557891 | 135.003498 |
| SRR18000098 | 45U | 44.2686111 | 6.12805556 | F | 0.02189731 | 126.690405 |
| SRR18000099 | 44U | 44.576533 | 6.33938802 | U | 0.00941603 | 158.87931 |
| SRR18000100 | 43U | 44.576533 | 6.33938802 | U | 0.01742602 | 210.030235 |
| SRR18000101 | 42U | 44.484026 | 6.42673097 | U | 0.01805332 | 93.0202399 |
| SRR18000102 | 41U | 44.38638 | 6.40138 | U | 0.01213298 | 1246.62694 |
| SRR18000103 | 40U | 44.38638 | 6.40138 | U | 0.01483673 | 101.001 |
| SRR18000104 | 3T | 44.22544 | 6.70695 | F | 0.00139046 | 72.0024988 |
| SRR18000105 | 39F | 44.34712 | 6.445 | F | 0.02695604 | 697.345577 |
| SRR18000106 | 38F | 44.34712 | 6.445 | F | 0.0137451 | 1434.86557 |
| SRR18000107 | 13T | 44.32732 | 6.26886 | F | 0.00107277 | 321.846327 |
| SRR18000108 | 37F | 44.3177778 | 6.44305556 | F | 0.00128231 | 1888.30185 |
| SRR18000109 | 36F | 44.3177778 | 6.44305556 | F | 0.00220538 | 1596.52224 |
| SRR18000110 | 35U | 44.3663889 | 6.41861111 | F | 0.00309868 | 1261.43003 |
| SRR18000111 | 34U | 44.3663889 | 6.41861111 | F | 0.00221412 | 875.766117 |
| SRR18000112 | 33F | 44.2988889 | 6.2725 | F | 0.00186129 | 163.902299 |
| SRR18000113 | 32F | 44.2988889 | 6.2725 | F | 0.00295822 | 150.722889 |
| SRR18000114 | 31U | 44.3769444 | 6.23555556 | F | 0.00386787 | 93.8985507 |
| SRR18000115 | 30U | 44.3769444 | 6.23555556 | F | 0.00190984 | 89.0369815 |
| SRR18000116 | 2T | 44.24837 | 6.71654 | F | 0.00094055 | 78.5114943 |
| SRR18000117 | 29U | 44.4097222 | 6.38083333 | U | 0.00191751 | 1870.34633 |
| SRR18000118 | 12T | 44.32732 | 6.26886 | F | 0.00110478 | 326.484758 |
| SRR18000119 | 28U | 44.4097222 | 6.38083333 | U | 0.0011166 | 1084.27711 |
| SRR18000120 | 27F | 44.23676 | 6.19958 | NA | NA | NA |
| SRR18000121 | 26F | 44.23676 | 6.19958 | NA | NA | NA |
| SRR18000122 | 25F | 44.266608 | 6.30630196 | F | 0.00782456 | 209.451024 |
| SRR18000123 | 24T | 44.2679 | 6.2099 | F | 0.00246582 | 284.596452 |
| SRR18000124 | 23T | 44.26506 | 6.21299 | F | 0.00178241 | 130.464768 |
| SRR18000125 | 22T | 44.26506 | 6.21299 | F | 0.00342109 | 69.1206897 |
| SRR18000126 | 21T | 44.26467 | 6.21779 | F | 0.00449693 | 149.426787 |
| SRR18000127 | 20T | 44.27347 | 6.20969 | F | 0.00449933 | 86.3830585 |
| SRR18000128 | 1T | 44.24132 | 6.70751 | F | 0.00130094 | 116.245627 |
| SRR18000129 | 11T | 44.31817 | 6.27965 | F | 0.00156749 | 91.4392804 |
| SRR18000130 | 10T | 44.321 | 6.27217 | F | 0.000954 | 619.356322 |

# 4 Parrot data

Sequencing data of the parrot *Psephotellus varius*.

Kerensa McElroy, Keira Beattie, Matthew R. E. Symonds & Leo Joseph (2018)
Mitogenomic and nuclear diversity in the Mulga Parrot of the Australian arid zone: cryptic subspecies and tests for selection
*Emu - Austral Ornithology*, **118**:1, 22-35, DOI: 10.1080/01584197.2017.1411765

| **SRR** | **McElroy ID** |
| --- | --- |
| SRR6214420 | B41207 |
| SRR6214421 | B4471 |
| SRR6214422 | B2117 |
| SRR6214423 | B22946 |
| SRR6214424 | B18697 |
| SRR6214425 | B20203 |
| SRR6214426 | B2966 |
| SRR6214427 | B32871 |
| SRR6214428 | B22948 |
| SRR6214429 | B28284 |
| SRR6214430 | R7511 |
| SRR6214431 | HLW99 |
| SRR6214432 | HLW77 |
| SRR6214433 | HLW90 |
| SRR6214434 | B49688 |
| SRR6214435 | B49747 |
| SRR6214436 | B46433 |
| SRR6214437 | B47715 |
| SRR6214438 | B9297 |
| SRR6214439 | HLW7303 |
| SRR6214440 | B51936 |
| SRR6214441 | B54105 |

# 5 Figure S1

Figure S1. Model fit of estimation accuracy explained by ‘nuclear genome size’ and ‘mapping depth of nuclear reads to the extranuclear reference’.
